# Chronic Stress and Gonadectomy Affect the Expression of Cx37, Cx40 and Cx43 in the Spinal Cord

**DOI:** 10.3390/life11121330

**Published:** 2021-12-01

**Authors:** Marija Jurić, Marta Balog, Vedrana Ivić, Benjamin Benzon, Anita Racetin, Ivana Bočina, Nives Kević, Suzana Konjevoda, Kálmán F. Szűcs, Róbert Gáspár, Marija Heffer, Katarina Vukojević, Sandor G. Vari, Natalija Filipović

**Affiliations:** 1 Department of Anatomy, Histology and Embryology, University of Split School of Medicine, Šoltanska 2, 21000 Split, Croatia; maarjur@gmail.com (M.J.); benjamin.benzon@mefst.hr (B.B.); anitamuic10@gmail.com (A.R.); kvukojev@gmail.com (K.V.); 2 Faculty of Medicine, Josip Juraj Strossmayer University of Osijek, Cara Hadrijana 10/E, 31000 Osijek, Croatia; marthab007@gmail.com (M.B.); vedrana.ivic@mefos.hr (V.I.); marija.heffer@gmail.com (M.H.); 3 Faculty of Science, University of Split, Ruđera Boškovića 33, 21000 Split, Croatia; bocina@pmfst.hr (I.B.); nkevic@pmfst.hr (N.K.); 4 Department of Health Studies, University of Zadar, Splitska 1, 23000 Zadar, Croatia; suzana.konjevoda@gmail.com; 5 Department of Pharmacology and Pharmacotherapy, Interdisciplinary Excellence Centre, University of Szeged, Dóm Tér. 12., H-6720 Szeged, Hungary; szucs.kalman@med.u-szeged.hu (K.F.S.); gaspar.robert@med.u-szeged.hu (R.G.); 6 International Research and Innovation in Medicine Program, Cedars–Sinai Medical Center, Los Angeles, CA 90048, USA; Sandor.Vari@cshs.org

**Keywords:** spinal cord, chronic stress, ovariectomy, orchidectomy, connexin

## Abstract

The study aimed to determine whether the exposure to chronic stress and/or performance of gonadectomy might lead to disturbance in the expression of connexin (Cx) 37, 40 and 43 in the spinal cord (SC), as a potential explanation for sex differences in stress-related chronic pain conditions. After the rats were sham-operated or gonadectomized, three 10-day sessions of sham or chronic stress were applied. Immunohistochemistry and transmission electron microscopy (TEM) were used to examine Cx localization and expression in the SC. The gonadectomy resulted in an increase of Cx37 expression in the dorsal horn (DH) of the female rats, but chronic stress suppressed the effects of castration. In male rats, only the combined effects of castration and chronic stress increased Cx37 expression. The influence of chronic stress on the DH Cx40 expression was inversely evident after the castration: increased in the ovariectomized female rats, while decreased in the orchidectomized male rats. We did not find any effect of chronic stress and castration, alone or together, on Cx43 expression in the DH, but the percentage of Cx43 overlapping the astrocyte marker glial fibrillary acidic protein (gfap) increased in the male stressed group after the castration. In conclusion, the association of the chronic stress with sex hormone depletion results in disturbances of the SC Cx expression and might be a possible mechanism of disturbed pain perception after chronic stress exposure.

## 1. Introduction

Chronic stress is frequently related to chronic pain conditions, such as fibromyalgia, irritable bowel syndrome, or neuropathic pain [1,2]. Pain conditions are neuroplastic alternations of the electrical properties that can cause increased excitability of neurons, as well as substantial changes in the permeability of the blood-brain barrier (BBB) [3,4,5]. Epidemiological studies and systematic reviews have pointed out a higher incidence of chronic pain conditions in women, but there is not enough information about the mechanisms underlying these sex-specific differences [6,7,8]. Sex hormones are important modulators of neuronal circuitries. Oestrogen influences the propagation of stress-associated visceral hypersensitivity and has been proposed as a basis for various chronic pain conditions [9,10], while testosterone inhibits the hypothalamic–pituitary–adrenal axis response [11] providing a possible explanation for a lower prevalence of some pathological conditions related to chronic stress in men [1]. In addition, sex hormones modulate the permeability of the BBB [3]. The BBB is a highly selective and dynamic physical divider of the circulatory system and neural tissue, heavily susceptible to local changes [12]. Its sole purpose is to maintain the homeostasis and proper functioning of the central nervous system (CNS) [13]. The barrier consists of endothelial cells, astrocyte end-feet, pericytes, perivascular microglia, and basal lamina. The communication between different cellular compartments in the BBB is enabled via gap junctions [12].

Gap junction intercellular communications (GJIC) in the CNS are known as electric synapses [14]. They are composed of two opposed hexameric formations of six connexins (Cxs) on each participating cell known as connexon [15,16]. By interconnecting the cytoplasm of two adjacent cells, the channel function of GJIC provides a robust synchronization of electrical activity between groups of neurons, glia, or between each other [17]. In addition, GJIC also mediates in the exchange of small-molecule metabolites such as ATP and glutamate [14,15,18]. Moreover, nonpaired connexons also known as hemichannels found on nonjunctional regions of the cell membranes, provide leakage of cytosolic molecules in the extracellular medium and vice versa under certain conditions [19,20]. Non-channel functions of Cxs include intracellular cascade signaling and modulation of cellular adhesion properties [14]. The Cx family of proteins is encoded by 20 genes in rodents and 21 genes in humans, and each protein is named according to its molecular weight [21]. Most of the Cxs are selectively expressed on specific cell groups with the greatest diversity in the CNS [18]. The information about the regulation on which GJIC depends is fairly scarce. For instance, they can be modulated by an entire spectrum of activity-dependent mechanisms such as glutamate ionotropic or metabotropic receptors, other signaling systems, and intracellular changes of pH and Ca2+ [15,22,23]. It is considered that the distinctive remodeling of Cx expression and function is of great importance for the genesis of pathological conditions in the CNS [14].

In pain perception and processing, spinal Cx expression and function seem to play a key role in the pathogenesis of neuropathic pain states induced by peripheral nerve or spinal cord injury, as well as in inflammatory and cancer-related pain conditions [24,25,26,27]. By using the water avoidance stress model in rats, Golovatscka et al. found an increase in spinal pro-inflammatory mediators and blood-SC-barrier (BSCB) permeability in stressed animals, alongside significant changes of the spinal Cx43 expression [4]. Considering the influence of sex hormones on Cxs expression and function in the CNS [28,29], changes in their quantity may be related to the higher incidence of chronic pain conditions in women related to chronic stress. Therefore, we hypothesized that sex and gonadectomy alone, or in combination with chronic stress, might lead to changes in the expression of different types of spinal Cxs. These changes could provide a potential explanation for sex differences in stress-related chronic pain conditions.

## 2. Materials and Methods

### 2.1. Animal Experiments

**Figure 1:**
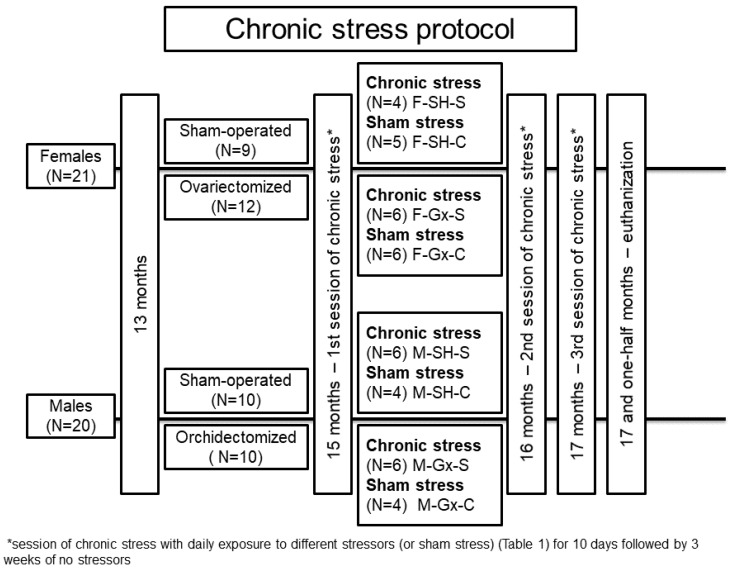
Flowchart presenting animal groups and chronic stress protocol. F-SH-S—a group of female, sham-operated stressed animals; F-SH-C—a group of female, sham-operated non-stressed animals; F-Gx-S—a group of female, ovariectomized stressed animals; F-Gx-C—a group of female, ovariectomized non-stressed animals; M-SH-S—a group of male, sham-operated stressed animals; M-SH-C—a group of male, sham-operated non-stressed animals; M-Gx-S—a group of male, orchidectomized stressed animals; M-Gx-C—a group of male, orchidectomized non-stressed animals.

Forty-one Sprague Dawley rats (21 females and 20 males) were used in the study (Figure 1). Animals were housed in standard cages with two animals per cage, at standard laboratory conditions: room temperature (21 ± 2 °C), the humidity of 40–60%, rat chow and water ad libitum, and a 12 h light/12 h dark cycle. At the age of 13 months, gonadectomy (Gx) was performed. The rats were anesthetized with isoflurane (Forane^®^, Abbott Laboratories Ltd., Queenborough, UK). One group of female rats (N = 12) was ovariectomized, one group of male rats (n = 10) was orchidectomized, and the other animals were sham-operated (SH). Ovariectomy of female rats was made by a midline abdominal incision [30], while male rats were castrated according to the method of Idris [31]. Two months after the surgery (age 15 months), all stress caused by surgery was considered irrelevant based on previous research where it was described that at least 4 weeks are required between surgery and exposure to chronic stress protocol [32]. The distribution of animals in experimental groups is presented in Figure 1. Three 10-day sessions of chronic stress or sham stress were applied. A different stressor was administered on each day of the session, with a 3-week break between sessions. The stress protocol has been optimized through previous experiments and published studies [32,33,34]. The sham stress group was exposed to the same environment as the chronic stress group but without the stressors. Stressors were cold restraint (60 min in metal tubes at +4 °C), light overnight, noise overnight (alarms on the phone at irregular intervals), swim test (swimming in cold water for 3 min), cage rotation (on laboratory shaker, 3 min per rat), glucose tolerance test (GTT) measurement and 12 h of food deprivation (Table 1). GTT as a stressor was performed only in the chronic stress group. Protocol for GTT was previously published [35]. Animals were sacrificed at the age of 17.5 months and the cranial thoracic SC tissues (Th1-Th3) were collected for analysis. Experiments were carried out at the Animal Facility of the Faculty of Pharmacy, University of Szeged, and approved by the National Scientific Ethical Committee on Animal Experimentation in Hungary (approval number: IV./3796/2015).

In order to confirm the effect of stress exposure, changes in body weight were followed in addition to the glucose tolerance test (only in stress-exposed animal groups). Moreover, the behavioral activity cage test (Ugo Basile, Varese, Italy; cat number 47420) was performed in the chronic stress group only, to confirm the development of anxiety/depressive-like phenotypes. The test was performed a day before the first GTT in the 1st stress session (control reference, baseline measurement), and a day after the last stress in the 1st, 2nd, and 3rd stress session (chronic stress and prolonged chronic stress reference). Rats were individually placed in the device box and allowed to move freely. Vertical and horizontal movements were recorded after 1 and 4 min. The device was cleaned with 70% ethanol between animals to reduce the olfactory distraction.

### 2.2. Immunohistochemical Staining

SC tissue samples were dissected, fixed in 4% paraformaldehyde in phosphate buffer saline (PBS), and dehydrated in graded ethanol. Afterward, the samples were embedded in paraffin wax, serially sectioned (5 µm), and mounted on glass slides. Sections were deparaffinized in xylene, rehydrated in ethanol and water, and treated with a heated citrate buffer for 30 min as previously described [36,37].

After washing in PBS and protein block application (30 min; ab64226, Abcam, Cambridge, UK), sections were incubated at room temperature in a humid chamber overnight with primary antibodies. Three sections of each animal were used for the immunohistochemical staining with at least 50 µm distances between sections. The first set of sections was incubated with goat anti-Cx43/GJA1 (1:300, ab87645, Abcam, Cambridge, UK) and mouse anti-GFAP (2E1) (1:50; sc-33673, Santa Cruz Biotechnology Inc., Santa Cruz, CA, USA). The second set was incubated with rabbit anti-Cx40/GJA5 (1:100, ab213688, Abcam, Cambridge, UK) and goat anti-GFAP antibody (1:100, ab53554 Abcam, Cambridge, UK). The third set was incubated with rabbit anti-Cx37/GJA4 (1:100, ab181701, Abcam, Cambridge, UK) and mouse anti-GFAP antibody (1:50). These were commercially available antibodies that have been used previously in immunohistochemistry in several publications (Table S1). After washing in PBS the secondary antibodies were applied for 1 h, with every set of sections receiving an appropriate mix of antibodies (all from Jackson ImmunoResearch Laboratories, Inc., West Grove, PA, USA): donkey anti-goat IgG (H + L) Alexa Fluor 488 conjugated antibody (1:400, 705-545-003), donkey anti-rabbit IgG (H&L) Alexa Fluor 488 conjugated antibody (1:400, 711-545-152), donkey anti-goat IgG (H + L) Rhodamine Red™-X (RRX) AffiniPure (1:400, 705-295-003) and donkey anti-mouse Rhodamine X conjugated antibody (1:400, 715-295-151). After final washing in PBS, nuclei were stained with 4′6′-diamidino-2-phenylindole (DAPI) solution for 2 min, and slides were cover-slipped. The exclusion of the primary antibody resulted in no staining of the tissue (Figure S1).

### 2.3. Data Acquisition and Immunohistochemical Analysis

The sections were observed under a fluorescence microscope (Olympus BX61, Tokyo, Japan). Images were captured using a cooled digital DS-Ri2 digital camera (Nikon, Tokyo, Japan) with NIS-Elements F software. The photomicrographs were taken at an objective magnification factor of ×40 and ×100 to capture the dorsal horn of SC and the area around (and including) the CC. The raw photomicrographs were processed by ImageJ software (National Institutes of Health, Bethesda, MD, USA). First, the fluorescence leakage reduction was performed for the green staining pictures through subtraction of red counter-signal for green fluorescence and then the median filter was used with a radius of 2.0 pixels. The red staining gfap-pictures were exposed only to the median filter with a radius of 5.0 pixels. Subsequently, each picture was adjusted to the threshold method with a triangle thresholding algorithm and analyzed to measure the fluorescence percentage area. Finally, analysis of overlapping regions obtained by dual immunohistochemistry was carried out by merging the green and red staining threshold pictures and calculating the fluorescence percentage area of the overlapping part using Adobe Photoshop (Adobe Inc., San Jose, CA, USA). For the purpose of publication, subtraction of the background was performed and all the figures are slightly contrasted.

### 2.4. Tissue Preparation for Transmission Electron Microscopy (TEM)

Using an immunogold immunohistochemistry protocol tissues were prepared for TEM [38,39,40]. The fixation of the samples was carried out with 4% paraformaldehyde in PBS, after which samples were washed in PBS. Samples were cut with a vibratome (Vibratome Series 1000, Pelco 101, Ted Pella, Inc., Redding, CA, USA) into 20 µm thick sections. After washing in PBS, sections were incubated first in 50% ethanol for permeabilization and then in primary antibody for 48 h at +4 °C: rabbit anti-Cx37/GJA4 (1:100, ab181701, Abcam, Cambridge, UK); rabbit anti-Cx40/GJA5 (1:100, ab213688, Abcam, Cambridge, UK); and goat anti-Cx43/GJA1 (1:300, ab87645, Abcam, Cambridge, UK). Next, sections were rinsed in PBS after which overnight incubation followed with gold-conjugated donkey anti-rabbit or anti-goat secondary antibody (1:1000, 711-205-152 and 705-185-147, both from Jackson ImmunoResearch Laboratories, Inc., West Grove, PA, USA). The size of the gold particles used was 12 nm for anti-rabbit and 4 nm for anti-goat antibodies. On the next day, sections were rinsed in PBS, post-fixed in 1% osmium tetroxide (1 h), and then dehydrated in ethanol and embedded in Durcupan ACM resin (Sigma-Aldrich Inc., St. Louis, Missouri, USA). The sections were observed with a transmission electron microscope (JEM JEOL 1400, Jeol Ltd., Tokyo, Japan).

### 2.5. Statistical Analyses

Mead’s resource equation was used for sample size estimation, where the degree of freedom is well above 20. Hence, the study power is above 80%. GraphPad Prism 8 software was used for statistical analyses (version 8.0.1 for Windows, GraphPad Software, San Diego, CA, USA). Normality of distribution was tested by using Shapiro-Wilk test of normality (Table S2). To determine significant differences among groups, three-way ANOVA with Welch correction for unequal variances was used (Figure S2). Statistical significance was considered at *p* < 0.05.

## 3. Results

**Figure 2:**
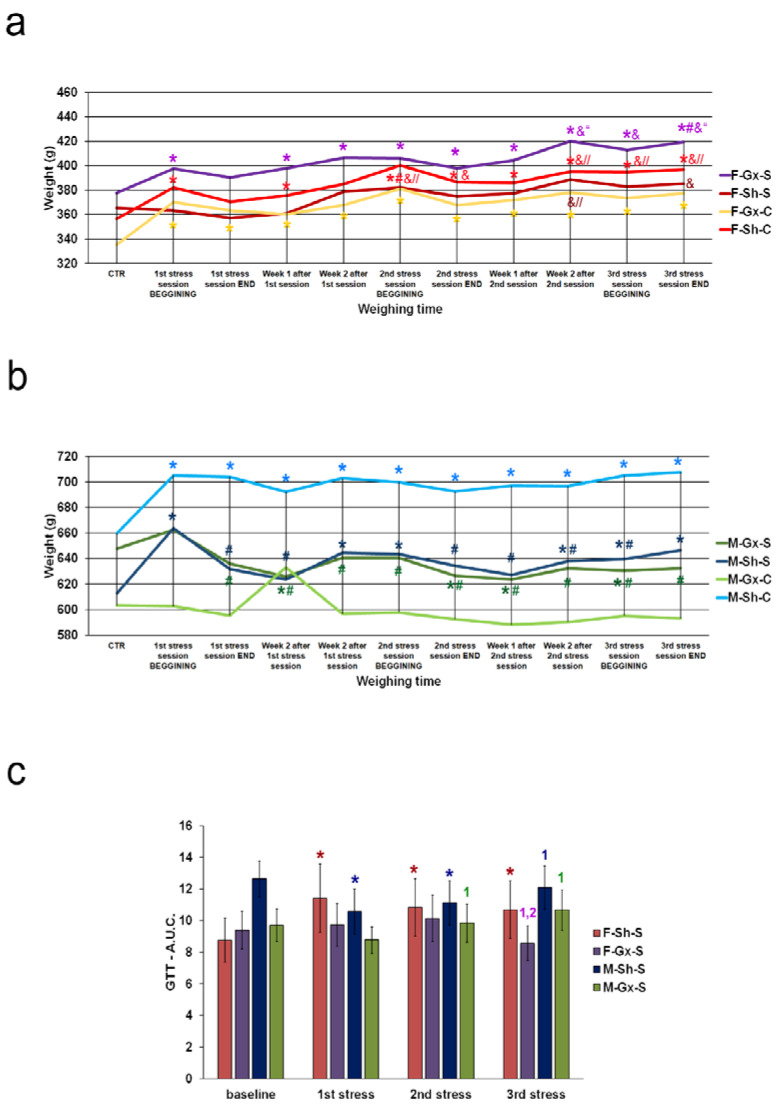
Body weight changes and glucose tolerance test results of rats after gonadectomies and chronic stress exposure. (**a**) body weights of female rats, (**b**) body weights of male rats; F-SH-S—a group of female, sham-operated stressed animals; F-SH-C—a group of female, sham-operated non-stressed animals; F-Gx-S—a group of female, ovariectomized stressed animals; F-Gx-C—a group of female, ovariectomized non-stressed animals; M-SH-S—a group of male, sham-operated stressed animals; M-SH-C—a group of male, sham-operated non-stressed animals; M-Gx-S—a group of male, orchidectomized stressed animals; M-Gx-C—a group of male, orchidectomized non-stressed animals. An asterisk denotes a statistically significant difference in comparison to the control measurement (CTR) *—*p* < 0.05; # denotes a statistically significant difference in comparison to the 1st stress session BEGINNING #—*p* < 0.05; &—denotes a statistically significant difference in comparison to the 1st stress session END &—*p* < 0.05; //—denotes a statistically significant difference in comparison to the week 2 after 1st stress session //—*p* < 0.05; “—denotes a statistically significant difference in comparison to the 2nd stress session BEGINNING “—*p* < 0.05. (**c**) Glucose tolerance test results of rats after gonadectomy and chronic stress exposure—area under the curve (A.U.C.) for blood glucose. An asterisk denotes a statistically significant difference in comparison to the baseline measurement *—*p* < 0.05; 1—denotes a statistically significant difference in comparison to the 1st stress measurement—*p* < 0.05; 2—denotes a statistically significant difference in comparison to the 2nd stress measurement—*p* < 0.05.

**Figure 3:**
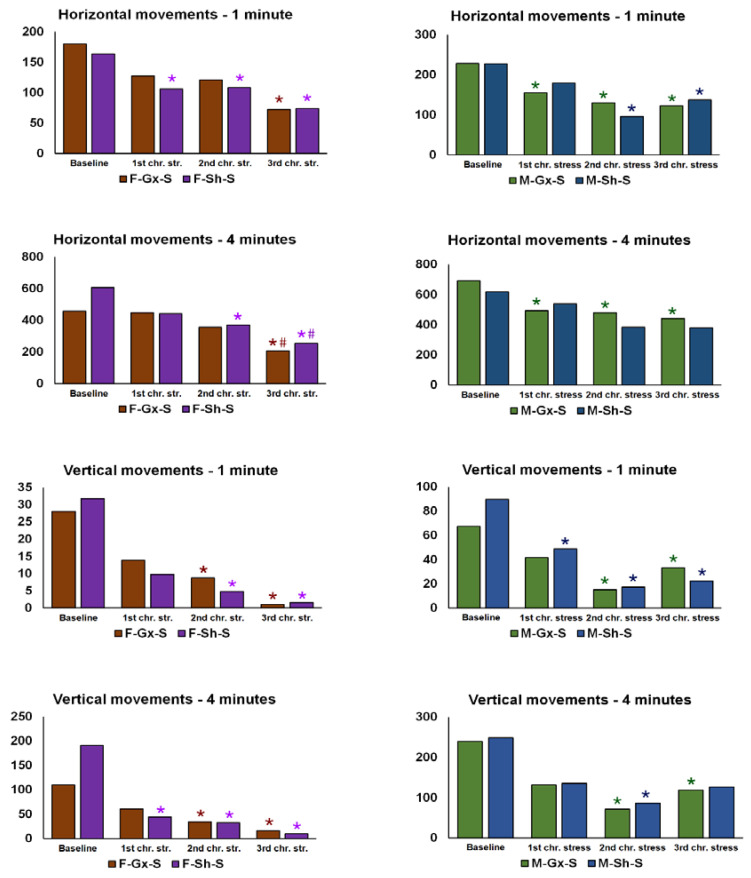
Results of the behavioral testing in stress-exposed groups of rats. F—female, M—male, Gx-S—gonadectomized chronic stress group, Sh-S—sham-operated chronic stress group. An asterisk denotes a statistically significant difference in comparison to the baseline measurement *—*p* < 0.05; # denotes a statistically significant difference in comparison to the measurement after the 1st chronic stress session.

We investigated the effects of sex, gonadectomies, and exposure to chronic stress on Cx37, Cx40, and Cx43 expression in the dorsal horn and around the central canal (CC; lamina X) of the SC. There were eight experimental groups: female sham-operated control (sham stress) (F-SH-C) and chronic stress groups (F-SH-S), female ovariectomized control (sham stress) (F-Gx-C) and chronic stress groups (F-Gx-S), male sham-operated control (sham stress) (M-SH-C) and chronic stress groups (M-SH-S), and male orchidectomized control (sham stress) (M-Gx-C) and chronic stress groups (M-Gx-S; Figure 1). The changes in body weight for all groups of animals in all phases of the experiment are shown in Figure 2. As a result of stress and/or gonadectomy, differences in body weight and its gain were found. In female rats, after gonadectomy or exposure to stress, the body weight did not increase additionally after 15 months of age. In the control group of animals (F-Sh-C), body weight continued to slowly increase until the end of the experiment. However, the stress in gonadectomized animals resulted in an even larger weight gain (Figure 2a). Male animals initially responded to stress with the absence of a normal weight gain, and they kept lower body weight until the end of the experiment Figure 2b). In addition, GTT results in sham-operated stressed female animals have, immediately after the first stress session, shown an increase in the area under the curve (A.U.C.; Figure 2c) for blood glucose, which was also apparent after the second and third stress session, indicating disturbed glucose handling. On the other hand, ovariectomy prevented this disturbance in the F-Gx-S group. M-Sh-S animals had the highest values of basal measurement of A.U.C. with a gradual decrease after the first and second stress protocol session and concomitant decrease in body weight. After the third stress session, A.U.C. increased to the high baseline level. In male orchidectomized rats, an increase in A.U.C. was evident after the second and third stress session (Figure 2c). Results of the behavioral testing are presented in Figure 3. A statistically significant drop in the total horizontal and vertical movements was found towards the end of the experiment in all groups exposed to the stress (male and female animals). The results of the three-way ANOVA comparisons of the connexin expression are presented in Table 2.

**Figure 4:**
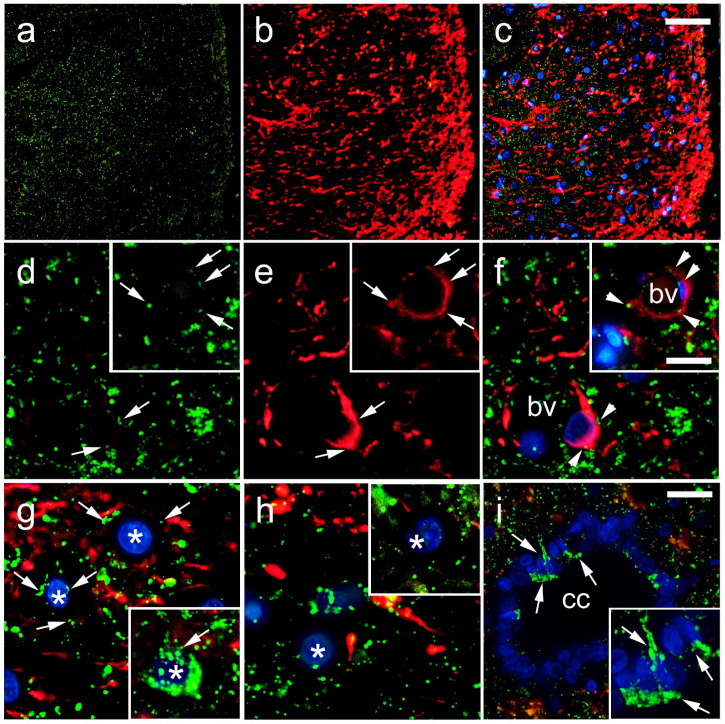
Expression of connexin 37 (Cx37) in the rat spinal cord. (**a**–**h**) Dorsal horn; (**i**) central canal (CC). Thoracic segments of the spinal cord were stained for Cx37 (green; arrows) and glial fibrillary acidic protein (gfap) (red; arrows). Blue—4′,6-diamidino-2-phenylindole (DAPI). (**a**–**c**) Lower magnification (scale bar on c = 50 µm); (**i**) scale bar = 20 µm; (**d**–**h**) and detailed inset on (**i**) higher magnification (scale bar on f = 10 µm). Arrowheads represent co-localization of Cx37 and gfap (yellow); asterisks show neuronal nuclei; bv denotes blood vessels.

**Figure 5:**
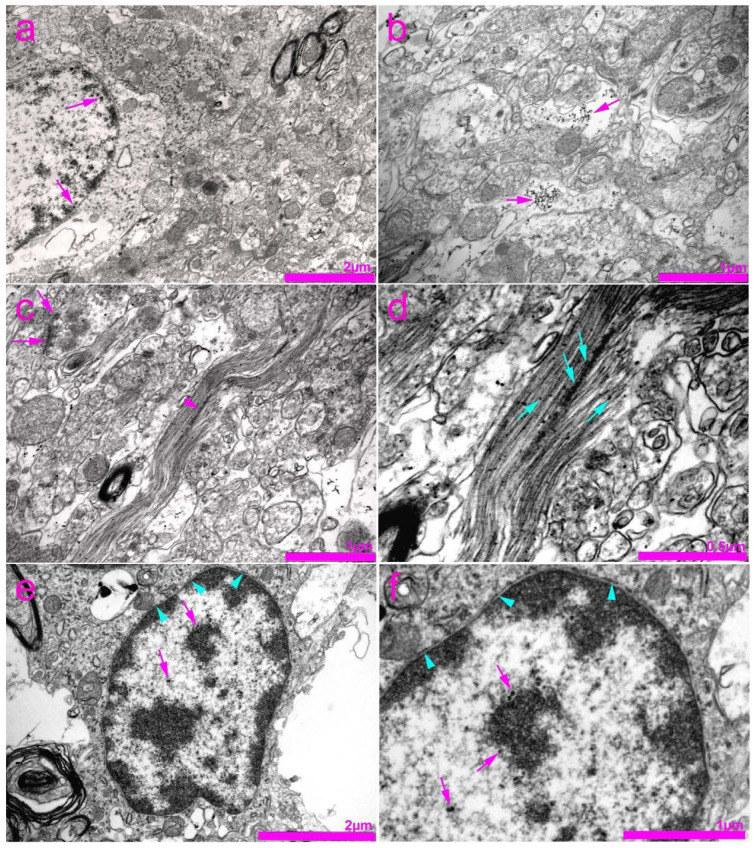
Transmission electron microscopy (TEM) photomicrographs of the rat spinal cord showing immunogold staining for connexin 37 (Cx37). (**a**) Expression of Cx37 in the nucleus of the neuron (arrows). (**b**) Expression of Cx37 in the cytoplasm of neurons (arrows). (**c**) Gap junctions (synapses) between two adjacent axon terminals (arrows). Expression of Cx37 in the area of neurofilaments (arrowhead). (**d**) Enlarged view of photomicrograph in panel (**c**) showing expression of Cx37 in the area of neurofilaments (arrows). (**e**) Expression of Cx37 in the nucleus of the neuron (arrows) and nuclear envelope (arrowheads). (**f**) Enlarged view of photomicrograph in panel (**e**) showing expression of Cx37 in the nuclear envelope (arrowheads) and nucleolar envelope (arrows).

In the histological sections of the SC, Cx37 showed the lowest expression, among the three investigated Cxs (Cx37, Cx40, Cx43). Strong immunoreactivity of Cx37 in the nuclei was apparent, especially in the nucleolar envelope, and scarcely, but observable in the cytoplasm of neurons (Figure 4g,h). Cx37 immunoreactivity was also present in the CC ependymal cells, as well as in the area surrounding the CC (lamina X; Figure 4i). Particularly strong immunoreactivity was observed in gfap-immunoreactive astroglia that encircled the blood vessels, but also in endothelial cells (Figure 4d–f). Immunogold staining for Cx37 and TEM confirmed the expression of Cx37 in the nucleus and nucleolus of neurons and their envelopes (Figure 5). In addition, expression of Cx37 was observed in the cytoplasm of the neurons, in the area of neurofilaments.

**Figure 6:**
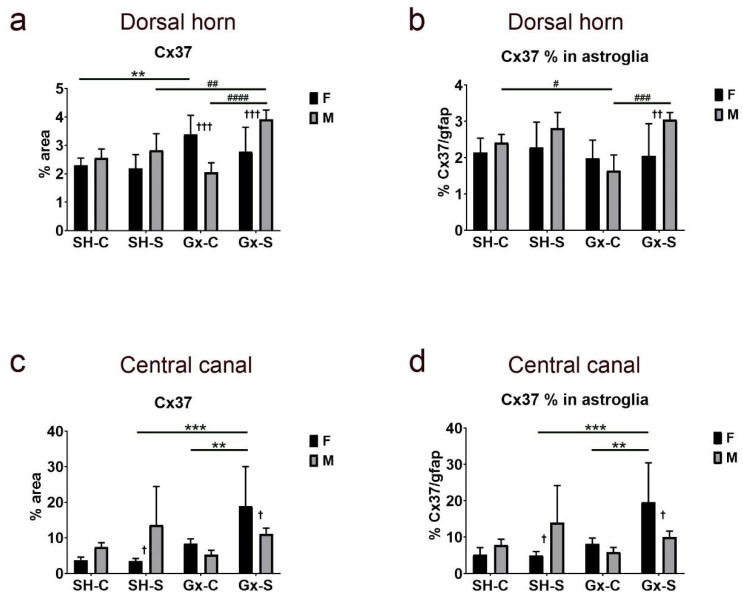
Changes in the expression of connexin 37 (Cx37) in the spinal cord of rats after gonadectomy and chronic stress exposure. Percentage area of immunofluorescence of Cx37 and Cx37/glial fibrillary acidic protein (gfap) co-localization in the dorsal horn ((**a**,**b**), respectively) and lamina X, including central canal (**c**,**d**). F—female, M—male, Gx-C—gonadectomized control (sham stress) group, Gx-S—gonadectomized chronic stress group, SH-C—sham-operated control (sham stress) group, SH-S—sham-operated chronic stress group. Asterisk denotes a statistically significant difference between female groups: **—*p* < 0.01, ***—*p* < 0.001; # denotes a statistically significant difference between male groups: #—*p* < 0.05, ##—*p* < 0.01, ###—*p* < 0.001, ####—*p* < 0.0001; † denotes a statistically significant difference between female and male groups: †—*p* < 0.05, ††—*p* < 0.01, †††—*p* < 0.001 between female and male groups.

Exposure to chronic stress alone did not have any significant effect on Cx37 expression in the dorsal horn, nor was a sex difference observed between sham-operated, non-stressed rats (Figure 6a). On the other hand, castration resulted in a significant increase of expression in the female rats, but chronic stress suppressed the effect of castration. In male rats, only the combined effect of castration and chronic stress resulted in an increase of Cx37 expression in the dorsal horn. The percentage of Cx37 immunoreactivity in gfap-immunoreactive astroglia in the dorsal horn of the SC did not significantly change in female animals due to castration and/or chronic stress exposure (Figure 6b) and it did not follow the course of change in total Cx37 (which increased after castration, as mentioned above). On the other hand, the course of change in Cx37 expression in gfap-immunoreactive astroglia in male animals was parallel to the change in total Cx37, with the lowest amount in orchidectomized rats (Figure 6b). Observed changes in Cx37 expression in castrated rats exposed to the chronic stress resulted in a male/female disproportion in total Cx37 expression (Figure 6a), as well as in the areas overlapping with gfap (Figure 6b). In the area around (and including) the CC, we found a significant change in total Cx37 expression (Figure 6c), as well as in co-localization with gfap only in female rats (Figure 6d), in which the expression significantly increased in animals exposed to both castration and chronic stress protocol.

**Figure 7:**
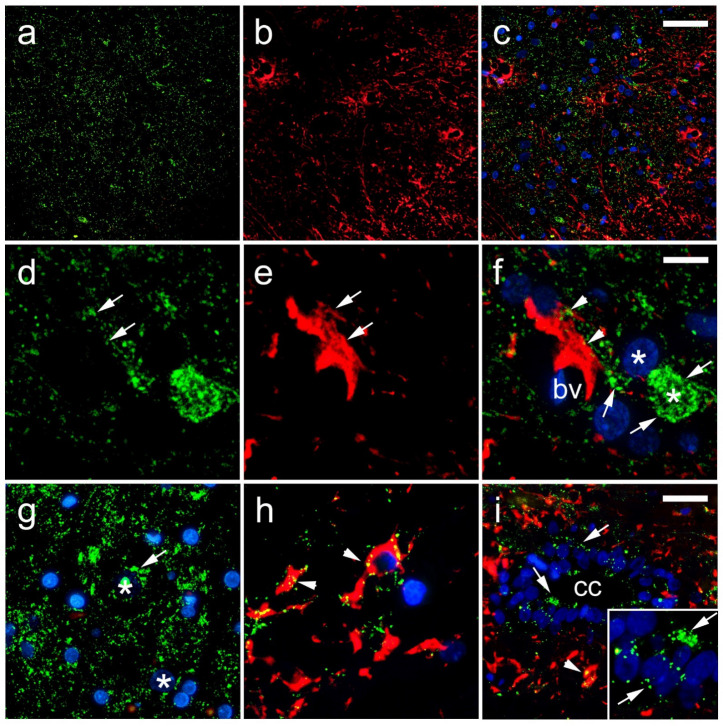
Expression of connexin 40 (Cx40) in the rat spinal cord. (**a**–**h**) Dorsal horn; (**i**) central canal (CC). Thoracic segments of the spinal cord were stained for Cx40 (green; arrows) and glial fibrillary acidic protein (gfap) (red; arrows). Blue—4′,6-diamidino-2-phenylindole (DAPI); (**a**–**c**) and (**g**)—lower magnification (scale bar on (**c**) = 50 µm); (**i**) scale bar = 20 µm; (**d**–**f**,**h**), and detailed inset on (**i**) higher magnification (scale bar on (**f**) = 10 µm). Arrowheads represent co-localization of Cx40 and gfap (yellow); asterisk in (**g**) shows neuronal nucleus; bv denotes blood vessels.

**Figure 8:**
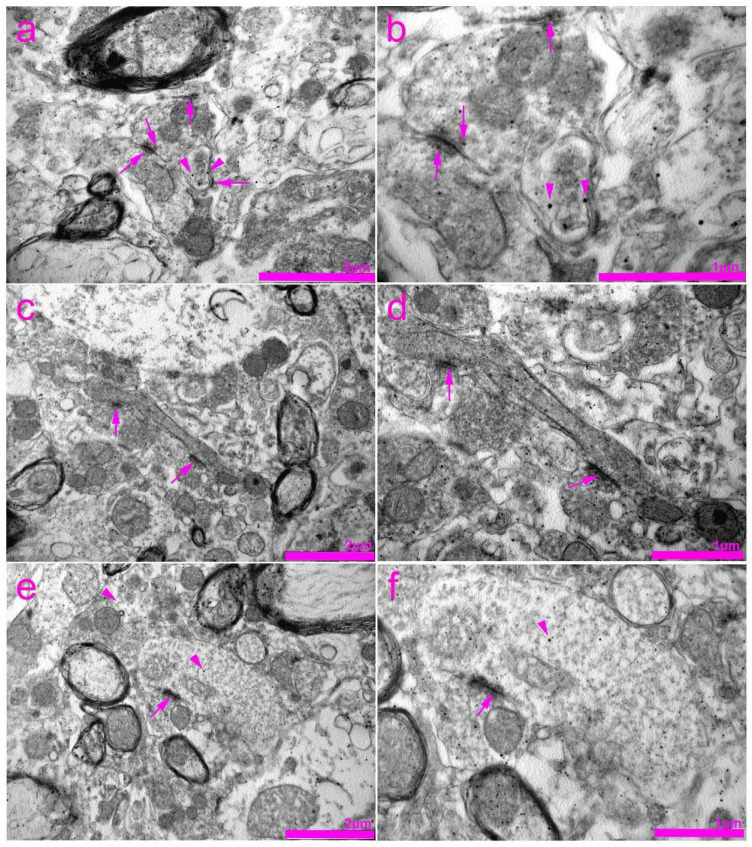
Transmission electron microscopy (TEM) photomicrographs of the rat spinal cord showing immunogold staining for connexin 40 (Cx40). (**a**) Expression of Cx40 in the cytoplasm of neurons (arrowheads) as well as in the axoaxonic synapses (arrows). (**b**) Enlarged view of the image in panel (**a**). (**c**) Expression of Cx40 in the area of axodendritic synapses (arrows). (**d**) Enlarged view of the image in panel (**c**). (**e**) Expression of Cx40 in the area of axoaxonic synapses (arrows) and cytoplasm of the neurons (arrowheads). (**f**) Enlarged view of the image in panel (**e**).

In the histological sections of the rat SC, we observed a strong immunoreactivity of Cx40 in the cytoplasm of neurons (Figure 7d–g). The overlapping region of Cx40 and gfap was found in astrocytes around (Figure 7g,i), and it was not related to the blood vessel walls (Figure 7f,h). A very strong Cx40 immunoreactivity was apparent in the ependymal cells of the CC. Unlike the Cx37 expression, a rare Cx40 immunoreactivity was seen in neuronal nuclei (Figure 7g,i and Figure 8). TEM confirmed the expression of Cx40 in the cytoplasm of the neurons (Figure 8). In addition, Cx40 was also observed in the axoaxonic and axodendritic synapses.

**Figure 9:**
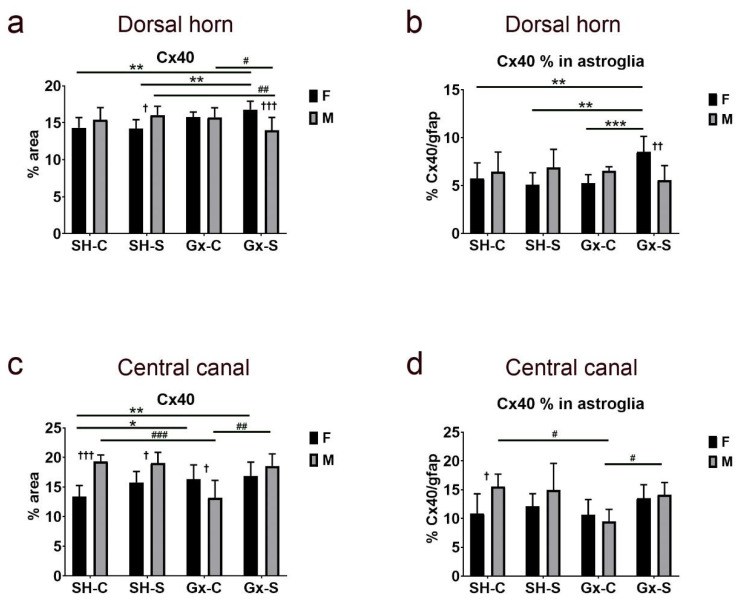
Changes in the expression of connexin 40 (Cx40) in the spinal cord of rats after gonadectomy and chronic stress exposure. Percentage area of immunofluorescence of Cx40 and Cx40/glial fibrillary acidic protein (gfap) co-localization in the dorsal horn ((**a**,**b**), respectively) and lamina X, including central canal (**c**,**d**). F—female, M—male, Gx-C—gonadectomized control (sham stress) group, Gx-S—gonadectomized chronic stress group, SH-C—sham-operated control (sham stress) group, SH-S—sham-operated chronic stress group. Asterisk denotes statistically significant difference between female groups: *—*p* < 0.05, **—*p* < 0.01, ***—*p* < 0.001; # denotes statistically significant difference between male groups: #—*p* < 0.05, ##—*p* < 0.01, ###—*p* < 0.001 between indicated male groups; † denotes statistically significant difference between female and male groups: †—*p* < 0.05, ††—*p* < 0.01, †††—*p* < 0.001.

The analysis of expression in the dorsal horn did not reveal the isolated effect of castration or chronic stress on the total amount of Cx40 expression or in areas overlapping with gfap, a marker for astroglia (Figure 9a,b). However, the influence of chronic stress on total dorsal horn Cx40 expression was evident after castration in both male and female animals, with an opposite direction of change in the two sexes: increasing significantly in ovariectomized female rats, while decreasing in orchidectomized male rats (Figure 9a). The expression of Cx40 in gfap significantly increased only in female ovariectomized rats; it did not show a significant change in male rats (Figure 9b). In addition, expression of total Cx40 around (and including) the CC significantly increased in both female castrated groups (stressed and non-stressed), when compared to the control (non-ovariectomized, non-stressed) groups (Figure 9c). Expression of total Cx40 in the CC area significantly decreased after orchidectomy in male rats, and this effect was suppressed after chronic stress exposure (Figure 9c). Furthermore, the percentage of Cx40 immunoreactivity overlapping the astrocyte marker gfap followed a similar trend to the total Cx40 expression: it significantly increased in female rats after a combined castration and chronic stress exposure, while in male rats it significantly decreased after the castration, but this effect was diminished after the chronic stress exposure (Figure 9d).

**Figure 10:**
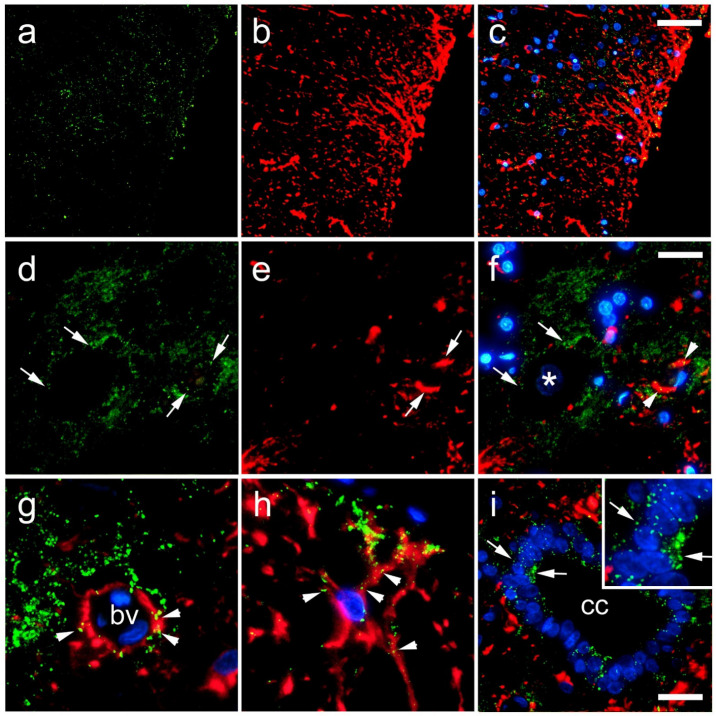
Expression of connexin 43 (Cx43) in the rat spinal cord. (**a**–**h**) Dorsal horn; (**i**) central canal (CC). Thoracic segments of the spinal cord were stained for Cx43 (green; arrows) and glial fibrillary acidic protein (gfap) (red; arrows). Blue—4′,6-diamidino-2-phenylindole (DAPI). (**a**–**c**) Lower magnification (scale bar on (**c**) = 50 µm); (**i**) scale bar = 20 µm; (**d**–**h**) and detailed inset on (**i**) higher magnification (scale bar on (**f**) = 10 µm). Arrowheads represent co-localization of Cx43 and gfap (yellow); asterisks show neuronal nuclei; bv denotes blood vessels.

**Figure 11:**
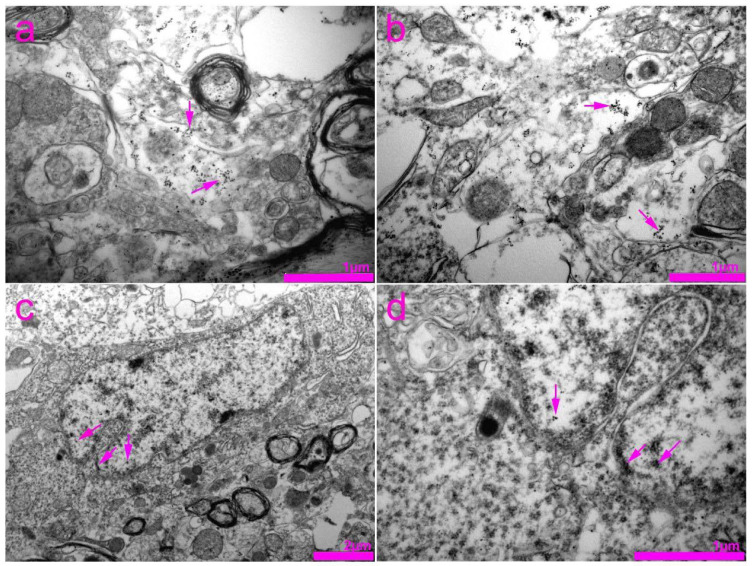
Transmission electron microscopy (TEM) photomicrographs of the rat spinal cord showing immunogold staining for connexin 43 (Cx43). (**a**,**b**) Expression of Cx43 in the cytoplasm of neurons (arrows). (**c**) Expression of Cx43 in the nucleus and area of the nuclear envelope (arrows) of the glia cell. (**d**) Enlarged view of the image in panel (**c**).

In the histological sections of the SC, we found immunoreactivity of Cx43 in gfap-immunoreactive astrocytes in general (Figure 10d–f,h), as well as around the blood vessels (Figure 10g). Unlike Cx37 and Cx40 expression, Cx43 immunoreactive dots were extremely rarely seen in neurons (Figure 10d–f; compare with Figure 4 (Cx37) and Figure 7 (Cx400). We also found Cx43 immunoreactivity in the ependymal cells of the CC (Figure 10i). TEM in combination with immunogold staining for Cx43 revealed a rare expression of Cx43 in the cytoplasm of the neurons (Figure 11). In addition, by using TEM, we found the expression of Cx43 in the nucleus and the area of the nuclear envelope of the glial cells.

**Figure 12:**
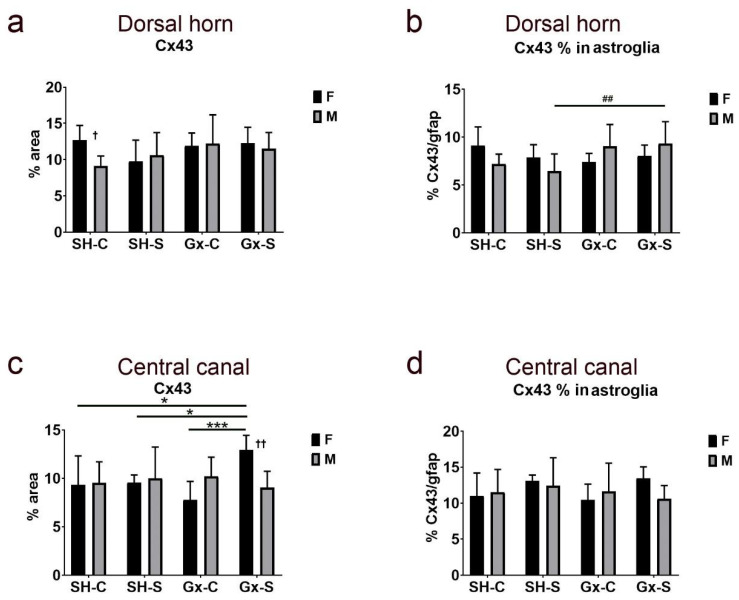
Changes in the expression of connexin 43 (Cx43) in the spinal cord of rats after gonadectomy and chronic stress exposure. Percentage area of immunofluorescence of Cx43 and Cx43/glial fibrillary acidic protein (gfap) co-localization in the dorsal horn ((**a**,**b**), respectively) and lamina X, including central canal (**c**,**d**). F—female, M—male, Gx-C—gonadectomized control (sham stress) group, Gx-S—gonadectomized chronic stress group, SH-C—sham-operated control (sham stress) group, SH-S—sham-operated chronic stress group. An asterisk denotes a statistically significant difference between female groups: *—*p* < 0.05, ***—*p* < 0.001; # denotes a statistically significant difference between male groups: ##—*p* < 0.01; † denotes a statistically significant difference between female and male groups: †—*p* < 0.05, ††—*p* < 0.01.

We did not find a significant effect of chronic stress and castration, either alone or together, on total Cx43 expression in the dorsal horn (Figure 12a). However, the expression of Cx43 in the dorsal horn was significantly higher in females compared to males in the control groups (sham-operated non-stressed). The female control group exposed to chronic stress showed a slight decrease in Cx43 expression, but it was not statistically significant. On the other hand, the percentage of Cx43 expression overlapping the astrocyte marker gfap was significantly higher in the male stressed group after castration (compared to sham-operated stressed rats) (Figure 12b). The expression pattern of Cx43 in the area encircling (and including) the CC in female animals followed a similar pattern of expression as for the other studied Cxs (Cx37, Cx40), being significantly increased after combined ovariectomy and chronic stress exposure (Figure 12c). The combination of castration and chronic stress also resulted in a higher expression of total Cx43 in female rats compared to male rats (Figure 12c).

## 4. Discussion

Gap junctions in the CNS represent an important element in intercellular communication that enables the maintenance of metabolic and electrical synchronism [15]. Plastic changes in the expression and function of Cxs, the building blocks of gap junctions, can lead to the development of various pathological conditions [12,14]. The roles, distribution, and organization of Cxs within neuronal circuitry of the pain processing areas in the SC are still largely unknown. In this research, changes in Cx expression in the dorsal horn of the SC after exposure to chronic stress and castration were studied, as a possible explanation for sex-specific differences in the incidence of chronic pain conditions. We studied the expression of glial Cx43 [41] and also Cx37 and Cx40 whose expression was previously found to be the most prevalent in endothelial cells of the CNS [5,12,18]; they were also described in neurons of the SC [42].

Data about Cx37 expression in the SC are scarce. Cx37 expression was reported in the SC of rats [42,43] and the mudpuppy (Necturus maculatus) [44] but without any details about its localization at the cellular level. In addition, a strong expression of Cx37 was found in the developing motor areas of the SC and motor neurons of adult rats [42]. The observed expression of Cxs in the nucleus is not new. Nuclear Cx expression has been previously related to the regulation of gene expression in control of growth and differentiation, by mechanisms independent of GJIC [45,46,47,48,49]. Although primarily related to the cell membrane [50], Cxs are also commonly found in the cytoplasm, in physiological and some pathological conditions [45,46,51], since the half-lives of Cxs are short, and they are being constantly synthesized and degraded. In addition to its expression in neurons, Cx37 immunoreactivity was found in gfap-immunoreactive astroglia that encircled the blood vessels. Previous studies found Cx37 occurrence in blood vessels mostly in vascular endothelium, but also monocytes/macrophages [52]. We also found Cx37 immunoreactivity in CC ependymal cells and the area surrounding the CC (lamina X).

Exposure to chronic stress alone did not cause changes in Cx37 expression in the dorsal horn. Ovariectomy resulted in an increase of Cx37 expression, while chronic stress suppressed this effect. In male rats, only the chronic stress combined with orchidectomy caused an increase of Cx37 expression in the dorsal horn. The pattern of change in Cx37-gfap co-localization was parallel to the change in total Cx37 only in male animals, with the lowest amount in orchidectomized rats. In the area around (and including) the CC only combined exposure to chronic stress and ovariectomy increased Cx37 expression in total and in areas overlapping with gfap. Increased nerve Cx37 expression was previously related to hyperexcitability after peripheral nerve injury [43]. Hence, it could be assumed that changes in Cx37 expression might also be the cause of change in the excitability of SC neurons of male and female rats exposed to chronic stress.

During the present study, we observed strong immunoreactivity of Cx40 in the cytoplasm of SC neurons confirmed by TEM. Our TEM data revealed the presence of Cx40 in the cytoplasm of neurons, as well as in axodendritic and axo-axonal synapses. Unlike Cx37 expression, we did not observe Cx40 in neuronal nuclei. Until now, the expression of Cx40 in SC neurons was studied only in motor neurons of rats during development and adulthood [42] and in a mouse knock-out model, through which its role of intercellular coupling in the formation of neuromuscular synapses was established [53]. Nevertheless, we found a strong Cx40 immunoreactivity in the cytoplasm of all observed motor neurons as well as numerous other neurons in the dorsal horn of the SC, with a similar pattern of immunoreactivity as in the previously mentioned study by Chang et al. [42] (granular cytoplasmic and membrane staining). Moreover, the Cx40 immunoreactivity also overlapped with gfap in astrocytes, observed around and non-related to blood vessel walls. Intense Cx40 immunoreactivity was found in the ependymal cells of the CC.

Castration or chronic stress separately did not cause changes in total Cx40 expression nor in Cx40 areas overlapping with gfap in the dorsal horn. However, in both male and female animals, we found combined effects of chronic stress and gonadectomy on total dorsal horn Cx40 expression. The direction of change was the opposite for the two sexes: when combined with chronic stress, ovariectomy caused an increase, while orchidectomy caused a decrease of Cx40 expression. The expression of Cx40 in gfap overlapping areas significantly increased only in female ovariectomized rats. In the area of the CC, gonadectomy resulted in an increase of the total Cx40 in female animals and a decrease of Cx40 (total and astroglial) in male rats. These results indicate that oestrogens might suppress, while, on the other hand, testosterone might increase Cx40 expression. Decreased Cx40 protein expression and the resulting pathological communication between cells has been previously related to inadequate astrocyte–neuron coupling, and vascular dysfunction in the CNS in diabetes [54]. Hence, we could speculate that the observed changes in spinal Cx40 expression caused by the combined effect of gonadectomy and chronic stress could result in disturbed somatic and visceral pain perception by similar mechanisms.

The Cx43 immunoreactivity in the SC of rats overlapped with the gfap-immunoreactive astrocytes in general, including those around the blood vessels. Rare Cx43 immunoreactive dots were seen in neurons. These findings are in agreement with previous data on the predominant occurrence of Cx43 in SC microglia and astrocytes, where it has an important role in their interaction and ion transfer [55]. Our TEM results also point to the rare occurrence of Cx43 in the neuronal cytoplasm. In agreement with the majority of previous studies [8,50], when comparing the expression of different Cxs, we also found a dominant presence of Cx43 in the SC. Immunoreactivity of Cx43 was also present in the ependymal cells of the CC.

Although the expression of Cx43 in the dorsal horn was higher in female rats, chronic stress or castration, alone or combined, did not change total Cx43 expression in the dorsal horn. However, the percentage of Cx43 expression overlapping the astrocyte marker gfap increased in the dorsal horn of male rats after combined exposure to chronic stress and castration. The expression pattern of Cx43 in the area around CC significantly increased only in female rats after combined ovariectomy and chronic stress exposure. The results of various studies point to the increase in astrocyte and microglial Cx43 expression in the SC in various inflammatory conditions, after stroke, as well as after SC injury and its role in the perpetuation of astrocyte activation, impulse transmission modulation and initiation of the inflammatory cascade [56,57]. All these events ultimately lead to chronic pain and nerve tissue damage [57].

Initially different amounts of Cx43 that we found when comparing male and female rats in the control groups are in agreement with the data about the influence of sex on the expression of Cx43 in cardiomyocytes [58]. Unlike our results, Golovatscka and collaborators found that the total amount of Cx43 in the whole SC region may have decreased after repeated exposure to the water avoidance test [4]. These differences could be explained by the usage of different stressors and the duration of chronic stress exposure.

Hormones in general play an important role in the function of nerve tissue. It is reported that sex hormones exert a neuroprotective role that influences the permeability of the BBB. BBB cells play a complex role, ensuring the separation of circulating substances within the blood from nerve tissue, thus providing a well-balanced microenvironment in the brain [3]. In addition to endothelial cells, this role is also provided by other associated cells, including astroglia and pericytes [59]. Cxs are one of the key elements for maintaining the functionality of the BBB [12]; through the intercellular cross-talk between cells involved in the maintenance of the barrier; through their effect on the tight-junction proteins, i.e., regulating BBB permeability [12,59,60] and their possible involvement in the release of pro-inflammatory mediators that influence the permeability of the BBB in pathological conditions [59]. The neuroprotective role of sex hormones is considered to be accomplished through the impact on endothelial cells by altering their functions, enhancing inter-endothelial cell tight junctions and providing anti-inflammatory responses that limit lymphocyte extravasation [61]. Sex hormone depletion caused by gonadectomy, therefore, leads to a greater BBB permeability [29,61,62,63]. According to our results, we could speculate this might partially be a result of disturbed Cx expression. Hence, the changes in Cx expression that we found in the SC of rats exposed to gonadectomy and chronic stress might cause the change in pain perception and also influence the function and integrity of the BBB.

On the other hand, stress stimuli cause activation of neuroendocrine systems as a basic response to any disturbance of homeostasis which results in a change in the composition of circulating plasma [11]. The neuroendocrine response includes the release of glucocorticoids. The role of glucocorticoids is considered anti-inflammatory and immunosuppressive when mediated by inflammation. However, in chronic stress conditions, with chronically augmented concentrations of glucocorticoids, it is considered that glucocorticoids increase the expression of pro-inflammatory factors [64,65]. Depending on the type of stimulus, the individual sensitivity and the CNS region studied, responses to chronic stress can eventually lead to overall changes in the CNS [5]. Results of our study have shown that the SC was more susceptible to stress after a gonadectomy, which caused additional changes in Cx expression.

All of the investigated Cxs have shown strong immunoreactivity in the ependymal cells of the CC. Ependymal cells of the CC are found to be a latent source of stem cells that reactivates after SC injury [66]. It was found that gap junctions containing Cx26 and Cx43 are important in the reactivation of ependymal proliferation [66] and SC neurogenesis [67]. In addition, Cx50 (whose level decreases after SC injury) [47] modulates the expression of Sox2 which is crucial for the self-renewal of neuronal stem cell pluripotency and conversion of glial cells in the neurons [68]. Taken together, our results could indicate a potential influence of chronic stress and gonadectomy on CC ependymal potential in SC regeneration.

Considering a central role of the dorsal horn superficial laminae in perception and modulation of pain [69] and that the spectrum of different roles attributed to SC lamina X neurons (around the CC) includes visceral nociceptive pathways [70], we can speculate that the changes in Cx expression that we found in these areas after combined exposure to chronic stress and gonadectomy may have roles in the maladaptation of somato- and viscerosensory pathways. Therefore, pharmacological modulation of gap junctions and other connexin functions could be a promising target in the treatment of painful conditions associated with chronic stress. However, it follows from these results that such a therapeutic approach should in any case be adapted to the sex and hormonal status of the patient. However, it will require a lot of additional research before Cx would become a druggable target for therapeutic purposes.

The main shortage of the present study is that, similar to many studies on connexin and pannexin, we used only immunohistochemistry for connexin and pannexin expression analysis. However, with Western blot and mass spectrometry spatial resolution is lost, which was of great importance in our research. Future studies should focus on using other methods such as testing the hormone replacement effect during the experiment, Western blot and mass spectrometry of the tissue in the SC, as well as the analysis of different pain processing areas (periventricular region of the fourth ventricle, trigeminal ganglia) in order to reveal whole complexity of the observed changes.

## 5. Conclusions

Although our study shows that chronic stress alone does not affect Cxs expression in the SC, its association with sex hormone depletion could lead eventually to disturbances in the SC Cx expression. Knowing the role of the Cxs in the CNS, we may assume that these changes might be one of the mechanisms that lead to disturbed pain perception after chronic stress exposure and its modulation might be a promising therapeutic strategy.

## Figures and Tables

**Table 1:** Daily stressors that were applied to rats in the chronic stress groups ^†^ during the three sessions of the chronic stress protocol.

**1st session of chronic stress protocol**	
**Day**	**Stressor**
1	Food deprivation (12 h)
2	GTT ^‡^
3	Cold restraint (+4 °C, 60 min) + food deprivation
4	GTT
5	Light overnight (09:00 pm–09:00 am)
6	Cage rotation (40 min)
7	Swim test
8	Noise overnight (09:00 pm–09:00 am)
9	Cold restraint (+4 °C, 60 min) + food deprivation
10	GTT
**2nd and 3rd sessions of chronic stress protocol**	
**Day**	**Stressor**
1	Cage rotation (40 min)
2	Swim test
3	Cold restraint (+4 °C, 60 min) + food deprivation
4	Noise overnight (09:00 pm–09:00 am)
5	Light overnight (09:00 pm–09:00 am)
6	Cage rotation (40 min)
7	Swim test
8	Noise overnight (09:00 pm–09:00 am)
9	Cold restraint (+4 °C, 60 min) + food deprivation
10	GTT

^†^ Chronic stress groups. ^‡^ Glucose tolerance test.

**Table 2:** Three-way ANOVA comparison of connexin expression in the spinal cord of rats.

		Cx37	Cx37/gfap	Cx40	Cx40/gfap	Cx43	Cx43/gfap
	Source of Variation			*p* Value			
**Dorsal horn**	**Stress**	**0.0496**	**0.0054**	0.9189	0.2921	0.6081	0.6437
	**Gonadectomy**	**0.0028**	0.1785	0.1829	0.3833	0.0901	0.1481
	**Sex**	0.3053	**0.0352**	0.9571	0.6437	0.3419	0.8565
	**Stress ∗ gonadectomy**	0.1258	0.1726	0.4519	0.191	0.7184	0.1896
	**Stress ∗ sex**	**0.0002**	**0.0238**	0.2346	0.1029	0.3124	0.9293
	**Gonadectomy ∗ sex**	0.1208	0.8167	**0.0016**	**0.036**	0.4869	**0.0065**
	**Stress ∗ gonadectomy ∗ sex**	**0.0048**	0.1147	**0.0382**	**0.0082**	0.1019	0.6697
**Central canal**	**Stress**	**0.0102**	**0.0103**	**0.0066**	**0.0424**	0.1149	0.179
	**Gonadectomy**	0.0647	0.1486	0.3542	0.1584	0.6144	0.6022
	**Sex**	0.7309	0.9857	**0.0087**	0.0809	0.7923	0.6254
	**Stress ∗ gonadectomy**	0.2145	0.2293	0.1796	0.087	0.2723	0.7958
	**Stress ∗ sex**	0.8294	0.8997	0.4357	0.9413	**0.049**	0.1564
	**Gonadectomy ∗ sex**	**0.0048**	**0.006**	**0.0006**	**0.0454**	0.4974	0.6823
	**Stress ∗ gonadectomy ∗ sex**	0.1827	0.0958	**0.0117**	0.3553	**0.034**	0.439

## Data Availability

The data presented in this study are available on request from the corresponding author.

## Supplementary Materials

The following are available online at https://www.mdpi.com/article/10.3390/life11121330/s1, Figure S1: Negative control, Figure S2: Exclusion of the primary antibody, Table S1: Antibody references, Table S2: Normality analysis.
