# Supplementary material for: Chronic Stress and Gonadectomy Affect the Expression of Cx37, Cx40 and Cx43 in the Spinal Cord
Source: Life (Basel). 2021 Dec 1;11(12):1330. doi: 10.3390/life11121330 (PMC8706389; doi:10.3390/life11121330)
Supplement: Supplementary file 1 [file life-11-01330-s001.zip › life-1393277-supplementary/life-1393277 -sup/Supplementary table 1.pdf]

Supplementary Table S1

Antibody	Publication	Method
ab87645	- Uribe P et al. Study on site-specific expression of bone formation and resorption factors in human dental follicles. Eur J Oral Sci 126:439-448 (2018).<br><br>- Shen Y et al. Connexin 43 SUMOylation improves gap junction functions between liver cancer stem cells and enhances their sensitivity to HSVtk/GCV. Int J Oncol 52:872-880 (2018).<br><br>- Shibata M et al. Involvement of glial cells in the autoregulation of optic nerve head blood flow in rabbits. Invest Ophthalmol Vis Sci 53:3726-32 (2012).<br><br>- Kosovic I et al. Spatio-temporal patterning of different connexins in developing and postnatal human kidneys and in nephrotic syndrome of the Finnish type (CNF). Sci Rep 10:8756 (2020).<br><br>- Kosovic I et al. Connexin Signaling in the Juxtaglomerular Apparatus (JGA) of Developing, Postnatal Healthy and Nephrotic Human Kidneys. Int J Mol Sci 21:8349 (2020).<br><br>- Juric M et al. Expression of Connexins 37, 43 and 45 in Developing Human Spinal Cord and Ganglia. Int J Mol Sci 21:9356 (2020).<br><br>- Lozic M et al. Alteration of Cx37, Cx40, Cx43, Cx45, Panx1, and Renin Expression Patterns in Postnatal Kidneys of Dab1-/- (yotari) Mice. Int J Mol Sci 22:1284 (2021).	Immunohistochemistry /Immunofluorescence
	- Zheng L et al. Phosphorylation of Cx43 residue Y313 by Src contributes to blocking the interaction with Drebrin and disassembling gap junctions. J Mol Cell Cardiol 126:36-49 (2019).	Western-blot
ab213688	- Wan HJ et al. Propofol-induced vasodilation of mesenteric arterioles via BKCa channel and gap junction. Exp Ther Med 16:2960-2968 (2018).<br><br>- Kosovic I et al. Spatio-temporal patterning of different connexins in developing and postnatal human kidneys and in nephrotic syndrome of the Finnish type (CNF). Sci Rep 10:8756 (2020).<br><br>- Kosovic I et al. Connexin Signaling in the Juxtaglomerular Apparatus (JGA) of Developing, Postnatal Healthy and Nephrotic Human Kidneys. Int J Mol Sci 21:8349 (2020).	Immunohistochemistry /Immunofluorescence

	- Lozic M et al. Alteration of Cx37, Cx40, Cx43, Cx45, Panx1, and Renin Expression Patterns in Postnatal Kidneys of Dab1-/- (yotari) Mice. Int J Mol Sci 22:1284 (2021).	
ab181701	- Jamalzaei P et al. Effects of Alginate Concentration and Ovarian Cells on In Vitro Development of Mouse Preantral Follicles: A Factorial Study. Int J Fertil Steril 13:330-338 (2020).<br><br>- Choi D et al. Piezo1 incorporates mechanical force signals into the genetic program that governs lymphatic valve development and maintenance. JCI Insight 4:e125068 (2019).<br><br>- Juric M et al. Expression of Connexins 37, 43 and 45 in Developing Human Spinal Cord and Ganglia. Int J Mol Sci 21:9356 (2020).<br><br>- Kosovic I et al. Spatio-temporal patterning of different connexins in developing and postnatal human kidneys and in nephrotic syndrome of the Finnish type (CNF). Sci Rep 10:8756 (2020).<br><br>- Kosovic I et al. Connexin Signaling in the Juxtaglomerular Apparatus (JGA) of Developing, Postnatal Healthy and Nephrotic Human Kidneys. Int J Mol Sci 21:8349 (2020).<br><br>- Lozic M et al. Alteration of Cx37, Cx40, Cx43, Cx45, Panx1, and Renin Expression Patterns in Postnatal Kidneys of Dab1-/- (yotari) Mice. Int J Mol Sci 22:1284 (2021).	Immunohistochemistry /Immunofluorescence
	- Sabry R et al. BPA and BPS Affect Connexin 37 in Bovine Cumulus Cells. Genes (Basel) 12:321. (2021).<br><br>- Ugwu N et al. Cutaneous and hepatic vascular lesions due to a recurrent somatic GJA4 mutation reveal a pathway for vascular malformation. HGG Adv 2:100028 (2021).<br><br>- Wang S et al. Contribution of Connexin Hemichannels to the Pathogenesis of Acute Lung Injury. Mediators Inflamm 2020:8094347 (2020).<br><br>- Choi D et al. Piezo1 incorporates mechanical force signals into the genetic program that governs lymphatic valve development and maintenance. JCI Insight 4:e125068 (2019).<br><br>- Lei R et al. Effects of Fullerenol Nanoparticles on Rat Oocyte Meiosis Resumption. Int J Mol Sci 19:699 (2018).	Western-blot
sc-33673	- Wang YF et al. Hyposmolality differentially and spatiotemporally	Immunohistochemistry

	modulates levels of glutamine synthetase and serine racemase in rat supraoptic nucleus. Glia 61:529-538 (2013).<br><br>- Torres A et al. Adenosine A3 receptor elicits chemoresistance mediated by multiple resistance-associated protein-1 in human glioblastoma stem-like cells. Oncotarget 7:67373-67386 (2016).<br><br>- Kim YE et al. Inhibitory effect of punicalagin on lipopolysaccharide induced neuroinflammation, oxidative stress and memory impairment via inhibition of nuclear factor-kB. Neuropharmacology 117:21-32 (2017).<br><br>- Miranda-Azpiazu P et al. A novel dynamic multicellular co-culture system for studying individual blood-brain barrier cell types in brain diseases and cytotoxicity testing. Sci. Rep. 8:8784 (2018).<br><br>- Jianrong S et al. DUSP14 rescues cerebral ischemia/reperfusion (IR) injury by reducing inflammation and apoptosis via the activation of Nrf-2. Biochem. Biophys. Res. Commun. 509:713-721 (2019).<br><br>- Ali W et al. Oral administration of a linoleic acid rescues Aβinduced glia-mediated neuroinflammation and cognitive dysfunction in C57BL/6N mice. Cells 9:667 (2020).	/Immunofluorescence
	- Wang YF et al. Hyposmolality differentially and spatiotemporally modulates levels of glutamine synthetase and serine racemase in rat supraoptic nucleus. Glia 61:529-538 (2013).<br><br>- Torres A et al. Adenosine A3 receptor elicits chemoresistance mediated by multiple resistance-associated protein-1 in human glioblastoma stem-like cells. Oncotarget 7:67373-67386 (2016).<br><br>- Kim YE et al. Inhibitory effect of punicalagin on lipopolysaccharide induced neuroinflammation, oxidative stress and memory impairment via inhibition of nuclear factor-kB. Neuropharmacology 117:21-32 (2017).	Western-blot
	- Miranda-Azpiazu P et al. A novel dynamic multicellular co-culture system for studying individual blood-brain barrier cell types in brain	Immunocytochemistry

	diseases and cytotoxicity testing. Sci. Rep. 8:8784 (2018).	
ab53554*<br><br>** It has been used in more than 230 published papers.<br>We mention only several more recent references.*	- Pretz D et al. Hyperleptinemia as a contributing factor for the impairment of glucose intolerance in obesity. FASEB J 35:e21216 (2021).<br><br>- Zhang Z et al. Downregulation of LncRNA Gas5 inhibits apoptosis and inflammation after spinal cord ischemia-reperfusion in rats. Brain Res Bull 168:110-119 (2021).<br><br>- Xu W et al. Melanocortin 1 receptor attenuates early brain injury following subarachnoid hemorrhage by controlling mitochondrial metabolism via AMPK/SIRT1/PGC-1a pathway in rats. Theranostics 11:522-539 (2021).<br><br>- Yao X et al. Neurotropin exerts neuroprotective effects after spinal cord injury by inhibiting apoptosis and modulating cytokines. J Orthop Translat 26:74-83 (2021).	Immunohistochemistry /Immunofluorescence
	- Hendrix RD et al. Alzheimer amyloid-ß- peptide disrupts membrane localization of glucose transporter 1 in astrocytes: implications for glucose levels in brain and blood. Neurobiol Aging 97:73-88 (2021).	Immunocytochemistry
