# Supplementary material for: Chronic Stress and Gonadectomy Affect the Expression of Cx37, Cx40 and Cx43 in the Spinal Cord
Source: Life (Basel). 2021 Dec 1;11(12):1330. doi: 10.3390/life11121330 (PMC8706389; doi:10.3390/life11121330)
Supplement: Supplementary file 1 [file life-11-01330-s001.zip › life-1393277-supplementary/life-1393277 -sup/Supplementary table 2.pdf]

Supplementary Table S2. Shapiro-Wilk test for normality of distribution

		F-SH-C	F-SH-S	F-Gx-C	F-Gx-S	M-SH-C	M-SH-S	M-Gx-NS	M-Gx-S
		Shapiro-Wilk test P value							
**Dorsal horn**	**Cx37**	0.5531	0.4664	0.6098	**0.0034**	0.6954	0.8128	0.5418	0.8664
	**Cx37/gfap**	0.6721	0.7550	0.8288	**0.0013**	0.5856	0.9709	0.2381	0.2642
	**Cx40**	0.2294	0.3121	0.6228	0.6882	0.8794	0.9355	0.7719	0.1780
	**Cx40/gfap**	0.8399	0.6911	0.9740	0.9104	0.8768	0.0831	0.7010	0.2696
	**Cx43**	0.1489	0.8357	0.6197	0.3557	0.5321	0.0590	0.0936	0.6510
	**Cx43/gfap**	0.3479	0.2837	0.6623	0.4158	0.2033	0.6404	0.9979	0.6754
**Central canal**	**Cx37**	0.4108	0.4809	0.3499	0.2489	0.7995	0.1241	0.6222	0.4769
	**Cx37/gfap**	0.2111	0.6312	0.4468	0.2542	0.9506	0.1557	0.5258	0.4016
	**Cx40**	0.1118	0.2926	0.3909	0.2927	0.1262	0.5104	0.5452	0.6251
	**Cx40/gfap**	0.0596	0.5025	0.6881	0.6348	0.0832	0.4997	0.7889	0.2470
	**Cx43**	0.6666	0.3184	0.9231	0.1170	0.4611	0.3460	0.7187	0.4336
	**Cx43/gfap**	0.8568	0.4083	0.8660	0.5714	0.3802	0.4243	0.7808	0.6769
