# Supplementary figures and images for: Chronic Stress and Gonadectomy Affect the Expression of Cx37, Cx40 and Cx43 in the Spinal Cord
Source: Life (Basel). 2021 Dec 1;11(12):1330. doi: 10.3390/life11121330 (PMC8706389; doi:10.3390/life11121330)

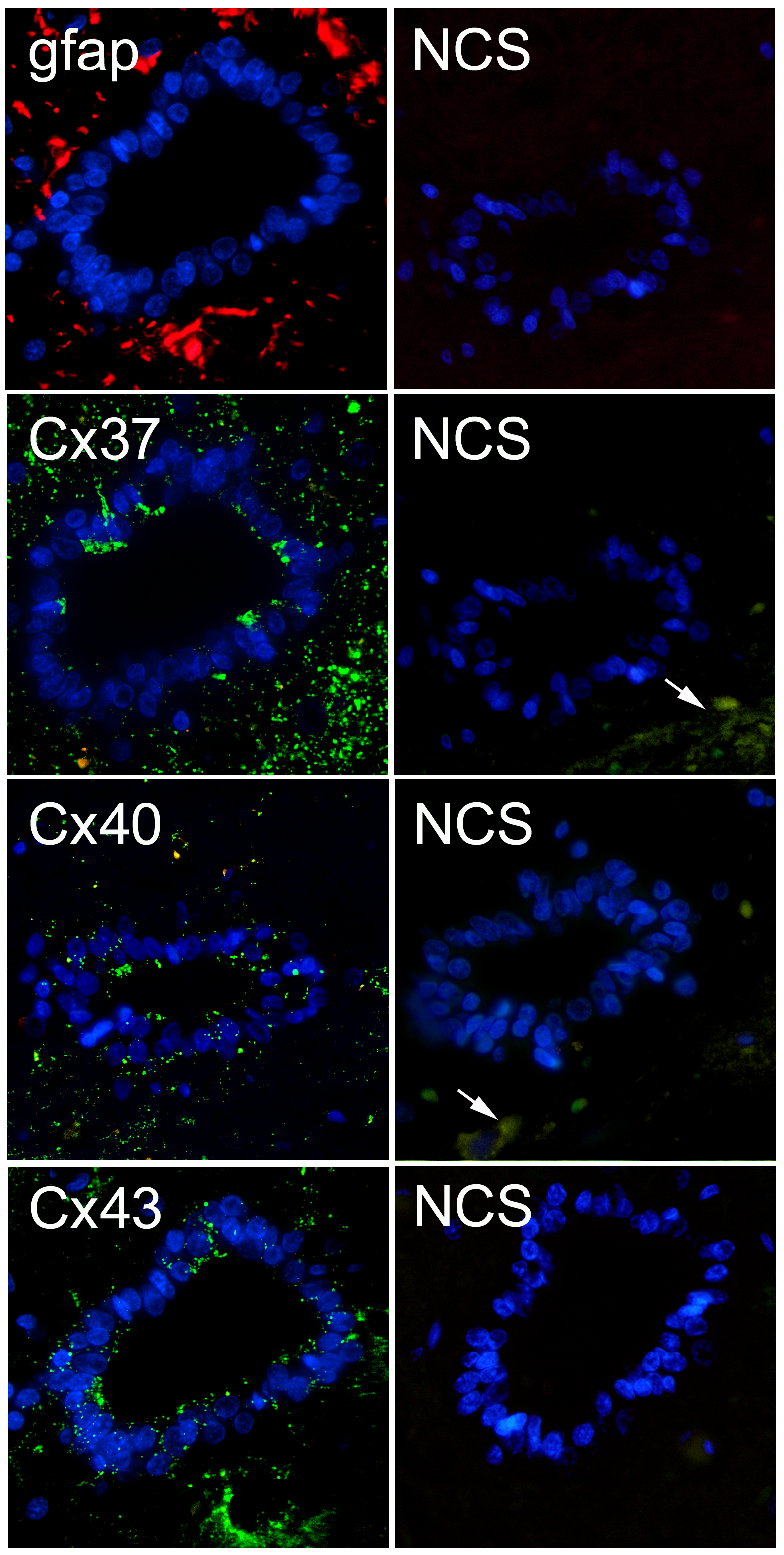

Supplement: Supplementary file 1 [file life-11-01330-s001.zip › life-1393277-supplementary/life-1393277 -sup/Suppl.Fig.1..tif]

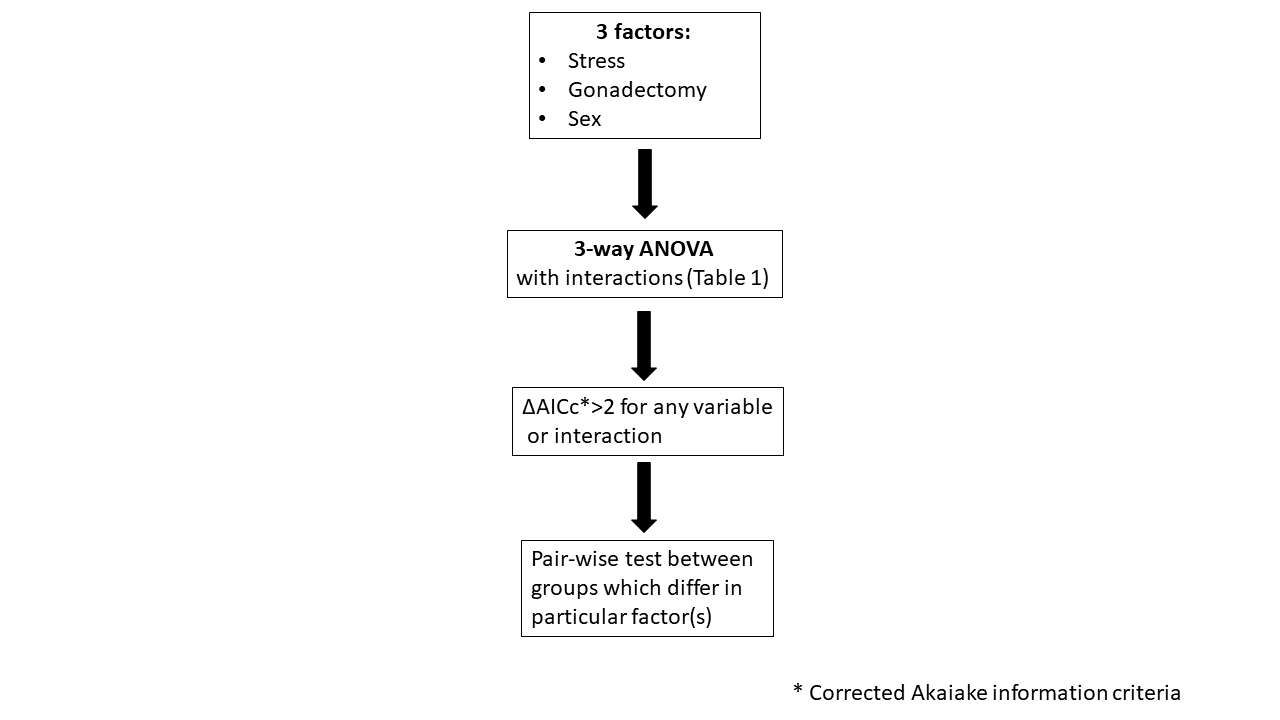

Supplement: Supplementary file 1 [file life-11-01330-s001.zip › life-1393277-supplementary/life-1393277 -sup/Suppl.Fig.2.png]
